# Tumor Endothelial Cells (TECs) as Potential Immune Directors of the Tumor Microenvironment – New Findings and Future Perspectives

**DOI:** 10.3389/fcell.2020.00766

**Published:** 2020-08-19

**Authors:** Laurenz Nagl, Lena Horvath, Andreas Pircher, Dominik Wolf

**Affiliations:** ^1^Department of Internal Medicine V (Haematology and Oncology), Medical University of Innsbruck, Innsbruck, Austria; ^2^Tyrolean Cancer Research Institute (TKFI), Innsbruck, Austria; ^3^Department of Oncology, Hematology, Rheumatology and Immunoncology, University Hospital Bonn (UKB), Bonn, Germany

**Keywords:** tumor microenvironment, tumor endothelial cells, immunoregulation, immunotherapy, antiangiogenic therapy

## Abstract

The tumor microenvironment (TME) plays a central role in cancer development and progression. It represents a complex network of cancer cell (sub-)clones and a variety of stromal cell types. Recently, new technology platforms shed light on the cellular composition of the TME at very high resolution and identified a complex landscape of multi-lineage immune cells (e.g., T and B lymphocytes, myeloid cells, and dendritic cells), cancer associated fibroblasts (CAF) and tumor endothelial cells (TECs). A growing body of evidence suggests that metabolically, genetically and on their transcriptomic profile TECs exhibit unique phenotypic and functional characteristics when compared to normal endothelial cells (NECs). Furthermore, the functional role of TECs is multifaceted as they are not only relevant for promoting tumor angiogenesis but have also evolved as key mediators of immune regulation in the TME. Regulatory mechanisms are complex and profoundly impact peripheral immune cell trafficking into the tumor compartment by acting as major gatekeepers of cellular transmigration. Moreover, TECs are associated with T cell priming, activation and proliferation by acting as antigen-presenting cells themselves. TECs are also essential for the formation of tertiary lymphoid structures (TLS) within the tumor, which have recently been associated with treatment response to checkpoint antibody therapy. Further essential characteristics of TECs compared to NECs are their high proliferative potential as well as greatly altered gene expression profile (e.g., upregulation of pro-angiogenic, extracellular matrix remodeling, and stemness genes), which results in enhanced secretion of immunomodulatory cytokines and altered cell-surface receptors [e.g., major histocompatibility complex (MHC) and immune checkpoints]. The TEC phenotype may be rooted in an aggressive tumor micro-milieu based on cellular stress *via* hypoxia and reactive oxygen species (ROS). *Vice versa* TECs might modulate TME immunogenicity thereby fostering cancer-associated immune suppression. This review aims to elucidate the currently emergent pathophysiological aspects of TECs with a particular focus on their potential role as regulators of immune cell function in the TME. It is a main future challenge to deeply characterize the phenotypic and functional profile of TECs to illuminate their complex role within the TME. The ultimate goal is the identification of TEC-specific drug targets to improve cancer (immuno-)therapy.

## Introduction

Cancer cells are tightly embedded within the tumor microenvironment (TME) and are in constant interaction with surrounding stromal cells, encompassing multi-lineage immune cells such as antigen-presenting cells (APC), T and B lymphocytes and myeloid cells, cancer-associated fibroblasts (CAFs) and endothelial cells (ECs). The TME is a rapidly growing field of interest and recent technological advances like single cell RNA sequencing (scRNAseq) were able to elucidate the TME heterogeneity in high resolution, defining cellular sub-clusters of stromal cell types by their transcriptomic profile, and further functional investigations are ongoing (Lambrechts et al., 2018). The TME exerts a direct effect on cancer cells and impacts cancer development and progression (Goveia et al., 2020). From a clinical perspective, a better understanding of the structural and, more importantly, the functional characteristics of the TME is essential, as many novel therapy approaches target the TME as immunotherapies as well as anti-angiogenic therapies. A central therapeutic focus lies upon reversing the intrinsic cancer-associated immune escape, which involves numerous innate and adaptive immune responses as well as tumor neo-angiogenesis. The latter is regulated not only by the cancer cell itself, but also by the surrounding cellular components of the TME (Galon and Bruni, 2020).

Under physiological conditions, the critical functions of ECs in immune surveillance and angiogenesis are well known and specified (Ley et al., 2007; Potente et al., 2011). Contrarily, characterization of tumor endothelial cells (TECs) has long been lacking behind due to technical difficulties in TEC isolation and limited options to single cell profile cell populations in depth. These hurdles have been overcome based on novel cell isolation protocols from primary human material as well as the advent of novel technology platforms, such as scRNAseq analysis including bioinformatics (Lambrechts et al., 2018; Goveia et al., 2020). Notably, TECs have a markedly altered morphologic and genetic phenotype including structural chromosomal changes and mutations when compared to normal endothelial cells (NECs). They exhibit stem cell-like origin thus being key to orchestrate tumor neo-angiogenesis (St Croix et al., 2000; Maishi et al., 2019). TECs serve as major gatekeepers for TME infiltrating immune cells and are actively involved in priming, activation or down-regulation of effector immune cells, thereby directly impacting anti-cancer immune responses (Buckanovich et al., 2008; Kambayashi and Laufer, 2014; Goveia et al., 2020). TECs also form a barrier to immune-stimulatory cells promoting the loss of protective anti-cancer immunity, a process referred to as “endothelial anergy” (De Sanctis et al., 2018). Furthermore, EC subpopulations contribute to the formation of tertiary lymphoid structures (TLS), which are essential for mounting an effective anti-tumor immunity (Sautès-Fridman et al., 2019). Moreover, lymphatic endothelial cells (LECs) are associated with lymphatic metastasis and represent an additional target to anti-angiogenic therapies (Hsu et al., 2019). A growing body of evidence suggests that TECs may be involved in tumor progression and metastasis (Maishi et al., 2019) implicating their possible prognostic and predictive potential.

The knowledge on TEC phenotypes is rapidly growing, however, their functional role in cancer-associated immune escape including response to established immune and anti-angiogenic therapies still needs to be specified in detail. In this review we will discuss the function of TECs within the TME and the tumor vasculature, with a particular focus on their regulatory and immune-modulatory properties from a pathophysiological perspective. A better understanding of TEC-associated deregulation of molecular pathways and alterations will potentially help to find novel therapeutic targets to evade TEC-mediated cancer immune escape and to enhance efficacy of already established (immune-activating) drugs. Key characteristics and important molecular factors associated with TECs are summarized in Tables 1, 2.

**TABLE 1:** Main characteristics of tumor endothelial cells (TEC) and tumor vessels.

Tumor vessels morphology	Tumor vessels represent with excessively branched and disorganized architecture. They are morphologically characterized by an irregular multi-layered endothelial lining, a discontinuous basement membrane and an inconsistent smooth muscle and pericyte sheath. These features result in an instable vessel wall promoting leakiness (fluid extravasation into the TME) and consequently higher interstitial pressures ultimately fostering tissue hypoxia and neo-angiogenesis (Klein, 2018; Tilki et al., 2007).
TEC morphology	TEC present with irregular cell surfaces and fenestrated cell walls. The intercellular junctions to neighboring cells, e.g., pericytes are loose and inconsistent (Morikawa et al., 2002). They may form multiple layered endothelia as compared to single layered NEC (Aird, 2012).
Metabolism	TEC are highly transcriptionally active and express higher RNA content compared to NEC (up to fourfold higher) (Lambrechts et al., 2018). Myc-target synthesis is highly enriched (Lin et al., 2012). TEC are hyper-glycolytic and largely use aerobic glycolysis for supplying their energy demands. Compared to NECS, they depict increased potential of self-renewal and are highly proliferative (De Palma et al., 2017).
Cytogenetics	TEC are characterized by chromosomal instability and abnormality, including aneuploic karyotypes, deletions, translocations or supernumerary centrosomes. These alterations might result in response to TME hypoxia and redox alterations, as well as epigenetic factors (e.g., methylations) (Hida et al., 2004; Deeb et al., 2011).
Molecular genetics	TEC show high intra-tumoral heterogeneity (St Croix et al., 2000) in terms of their gene expression profiles. Based on scRNAseq data, numerous TEC subtypes can be distinguished, such as tip and stalk EC (playing a major role in neo-angiogenesis) or postcapillary venous and activated postcapillary ECs (displaying immunoregulatory functions) (Lambrechts et al., 2018; Goveia et al., 2020). TEC are less responsive to pro-inflammatory stimulation (EC anergy). They can actively down regulate genes involved in immune cell homing (e.g., ICAM1 and VCAM1), chemostaxis (e.g., CXCL10 and CXCL11) as well as antigen presentation (e.g., HLA-DQB1 and HLA-DQA1) (Goveia et al., 2020). Furthermore, TEC may upregulate PD-L1 and PD-L2 to inhibit T cell activation via inhibitory immune checkpoints (Georganaki et al., 2018), express IDO 1 to promote local immunosuppression (Georganaki et al., 2020) and T cell apoptosis and upregulate Fas ligand expression inducing CD8+ T cell apoptosis (Motz et al., 2014).

**TME*, *tumor microenvironment; NEC, normal endothelial cell; scRNAseq, single cell RNA sequencing; PD-L1/2, programmed death receptor ligand 1/2; IDO 1, indoleamine-pyrrole 2,3-dioxygenase*.*

**TABLE 2:** Factors associated with tumor endothelial cells (TEC) and tumor vessels.

Molecule	Function
**Growth factors**	
VEGF superfamily	central angiogenic factors of tumor angiogenesis (Carmeliet and Jain, 2000; Potente et al., 2011). Induce genetic reprogramming of TECs, changes mode of immune cell interaction and adversely programs the TME (Albini et al., 2018; Maishi et al., 2019). Inhibits the up-regulation of leukocyte adhesion molecules (Griffioen et al., 1999; Dirkx et al., 2003; Flati et al., 2006).
VEGF-C	Central factor of lymphangiognesis (Hsu et al., 2019). Essential for HEV formation (Furtado et al., 2007). Overexpression fosters lymphatic metastasis (Decio et al., 2014; Wang et al., 2014; Yang et al., 2014; Goussia et al., 2018). Activated LECs by VEGF-C suppress tumor specific CD8+ T cells (Lund et al., 2012).
PDGF, HIF1, ANGPT2,	Central angiogenic factors of tumor angiogenesis (Carmeliet and Jain, 2000; Potente et al., 2011).
FGF2	Inhibits the up-regulation of adhesion molecules (ICAM and VCAM) (Griffioen et al., 1999; Dirkx et al., 2003; Flati et al., 2006).
bFGF	Angiocrine factor promoting tumor cell metastasis (Maishi et al., 2019).
EGFL7	Down-regulates adhesion molecules expression and fosters tumor vessel development via NOTCH signaling (Delfortrie et al., 2011; Nichol and Stuhlmann, 2012).
TGFβ	Angiocrine factor contributing to tumor metastasis (Maishi et al., 2019).
**Cytokines, interleukines, interferones, chemokines**	
TNFα	EC activation relies on TNFα and endothelial anergy is characterized by unresponsiveness to TNFα (De Sanctis et al., 2018; Georganaki et al., 2018). Enhances PD-L1 up-regulation on ECs (Georganaki et al., 2018)
IFNγ	Endothelial anergy is characterized by unresponsiveness to IFNγ (De Sanctis et al., 2018). Enhances PD-L1 upregulation on ECs (Georganaki et al., 2018).
IL-6, IL-8	Secreted by activated ECs mediating immune cell trafficking (Aird, 2012; Klein, 2018).
CXCL12/13, CCL19/21, IL-7	Chemokines/interleukines that are associated with the formation of HEVs and TLS (Sautès-Fridman et al., 2019).
CCL2/18, CXCL10/11	Attractant chemokines down-regulated by TECs to prevent immune cell trafficking, therefore mediating immune cell anergy (Griffioen et al., 1996; Huang et al., 2015; Lambrechts et al., 2018).
CXCL4	Chemokine strongly involved in leukocyte activation and endothelial rolling (Ley et al., 2007).
**Adhesion molecules**	
VCAM1, ICAM1 PECAM1, E/P-selectin	Expressed on ECs mediating leukocyte recruitments (Ley et al., 2007). Important gatekeepers of transmigration to the tumor compartment (De Sanctis et al., 2018). Multiple regulation mechanisms by tumor cells/TECs.
Soluble adhesion molecules	sCD146 and Endoglin inhibit TIL recruitment by competitive mechanisms and show direct VEGF-synergistic effects (Rossi et al., 2013; Stalin et al., 2016).
**Stemness factors, apoptosis-inducing/preventing molecules proteins**	
FASL	Expressed by TECs induces apoptosis in CD8^+^ T cells (Thommen and Schumacher, 2018).
Stemness molecules	TECs upregulate stem cell associated proteins (CD90, MDR1, ALP, Oct-4, and ALDH), which are associated with increased self renewal and high proliferative potential (Ohga et al., 2012; Ohmura-Kakutani et al., 2014; Hida et al., 2017)
**Immune checkpoints**	
PD-L1/2, TIM3	Expressed by TECs and promotes T cell arrest (Georganaki et al., 2018).
IDO1	Immune-regulatory molecule expressed by TECs in response to IFNγ restricting activation and inducing apoptosis of T cells (Georganaki et al., 2020).
**Antigen-presenting molecules**	
MHC I, II	Frequently expressed by ECs but lacking the co-stimulatory molecules (CD80 and CD86) (Shiao et al., 2007). MHC molecules can be down-regulated by TECs contributing to immune-evasion (Goveia et al., 2020).
**Other**	
NO	Affects leukocyte recruitment under malignant conditions (De Caterina et al., 1995). Directly suppresses effector T cells (De Sanctis et al., 2018). NO-antagonists restores T cell adhesion by up-regulation of adhesion molecules (Bouzin et al., 2007; Buckanovich et al., 2008).
ET1	Associated with ICAM1 expression and decreases TIL influx (Spinella et al., 2002; Buckanovich et al., 2008).
STING	Highly expressed on ECs of HEVs and impacts the expression of adhesion molecules (Demaria et al., 2015; Yang et al., 2019).
CLEVER1	Favors the selective influx of Treg and immuno-suppressive TAM (Nummer et al., 2007; Karikoski et al., 2014).
Biglycan	Preferentially expressed by high metastatic tumors and allows toll-like receptor expressing tumor cells to metastasize hematogenously (Maishi et al., 2016).
Galectins	Glycan-binding endogenous lectins which sustain tumor angiogenesis via autocrine and paracrine signaling (Elola et al., 2018). Directly interact with VEGFR2, bFGF, VEGFR3 and associated with resistance to anti-angiogenic therapies (Markowska et al., 2010; Markowska et al., 2011; Zhao et al., 2011; Laderach et al., 2013; Chen et al., 2016).
Jag1	Expressed by TECs to generate malignant vascular niche, which is associated with aggressive course and resistance to chemotherapy (Cao et al., 2016).
Slit2	Tumor-suppressive factor directly down-regulated by TECs via paracrine EphA2 signaling (Brantley-Sieders et al., 2011).

## Tumor Vessels: Evolution and Phenotype

Early on in tumorigenesis the sole diffusion of oxygen and nutrients from the surrounding tissue is not sufficient in supporting tumor cell growth. As cancer cells become hypoxic, they express angiogenic factors such as hypoxia inducible factor (HIF), vascular endothelial growth factor A (VEGFA), platelet-derived growth factor (PDGF) or angiopoietin 2 (ANGPT2) as well as proangiogenic chemokines and receptors to initiate neo-angiogenesis (a process termed “angiogenic switch”) (Carmeliet and Jain, 2000; Potente et al., 2011). Angiogenesis is the process of vessel formation from pre-existing vascular beds and is primarily guided by the proliferation of ECs. Binding of VEGFA to VEGF receptor 2 (VEGFR2) on ECs triggers the evolution of highly invasive navigating tip ECs and proliferating stalk ECs that lead to the formation and elongation of novel vascular sprouts by following the VEGFA gradient (Potente et al., 2011; De Palma et al., 2017). Fully formed tumor vessels express an immature phenotype, consisting of an instable vessel wall (inconsistent coverage of smooth muscle and pericytes), a discontinuous basement membrane and an irregular endothelial lining. The vessel architecture itself is excessively branched, chaotic and leaky (Tilki et al., 2007; Klein, 2018). These structural aberrations alter physiologic vessel functionality. Leakiness results in high intra-tumoral interstitial pressure (ITP) leading to poor perfusion and inconsistent blood flow in certain regions of the tumor, generating hypoxia and a shift to anaerobic glycolysis and acidosis (Cantelmo et al., 2016). This phenotype contributes to a pro-tumorigenic and immunosuppressive TME (Potente et al., 2011; Schaaf et al., 2018).

### Endothelial Cells: Structure, Function, and Autophagy

Endothelial cells are a highly heterogenous cell population, showing structural differences (e.g., direction of cell alignment within the epithelium, type of intercellular junctions, fenestrated vs. non-fenestrated) as well as a great genetic variability (Aird, 2012). They play a critical role in numerous physiological processes, including vascular stabilization and tonus control, hemostasis, angiogenesis as well as regulation of immune cell trafficking between blood and tissues, thus they are key in maintaining vessel homeostasis (Aird, 2012). In a healthy state ECs are typically found in a non-proliferating form (quiescence). However, they become activated on environmental stressors, such as pro-angiogenic signals and hypoxia to initiate angiogenesis, or inflammatory triggers (e.g., TNF and interleukins) to enhance immune cell trafficking. Activated ECs are able to up-regulate the expression of cell surface antigens (e.g., HLA molecules), adhesion molecules (selectins and integrins) and cytokines (e.g., IL-6 and IL-8) as well as to exhibit pro-thrombotic functions, ultimately resulting in a pro-immunogenic phenotype (Aird, 2012; Klein, 2018). Importantly, activated ECs have the ability to return quiescent once the activated stressor is removed. This functional plasticity is a key physiologic characteristic of NEC and is, at least partially, regulated by EC-intrinsic autophagy.

Autophagy comprises the mechanism of lysosomal degradation of potentially toxic cytoplasmatic compounds and is a major physiologic cytoprotective and pro-homeostatic cellular pathway. The molecular mechanisms of how autophagy mediates EC reactiveness and quiescence are complex, however, redox-homeostasis is a key determinant (Schaaf et al., 2019). In healthy and pre-malignant cells, autophagy depicts various onco-suppressive functions (reviewed in Rybstein et al., 2018), however, in cancer cells autophagy seems to be carcinogenic and tumor growth promoting, especially in hypoxic und nutrient-deprived areas of the TME (Schaaf et al., 2019). The functional mechanisms of autophagy in the tumor stromal cells, especially in TECs have not been clarified so far.

### Tumor Endothelial Cells

Tumor endothelial cells show distinct phenotypic differences on the molecular, structural, and functional level when compared to NECs (Table 1). Morphologically they present with irregular surfaces, excessively fenestrated cell walls and loose intercellular junctions to neighboring cells [e.g., pericytes (Morikawa et al., 2002)]. They may arrange in multiple layers (as opposed to single layer endothelial sheets in health vessels) which impedes proper function and promotes leakiness. On a molecular level, TECs are transcriptionally highly active, mirrored by an up to fourfold increase in RNA content when compared to NECs, potentially due to the high metabolic demands of nucleotide biosynthesis or glycolysis. Moreover, Myc-target synthesis is highly enriched in TECs, which is a well-recognized transcriptional factor associated with tumor-aggressiveness and known to impact angiogenesis (Lin et al., 2012; Lambrechts et al., 2018).

Tumor endothelial cells depict a wide inter- and intra-tumoral heterogeneity and already around 20 years ago TEC specific genes, called tumor endothelial markers (TEMs), were identified with, however, unclear function (St Croix et al., 2000). Only recent technological advances in RNA profiling by applying single cell transcriptome sequencing analysis (sc-RNA-seq) have allowed to characterize small cellular subpopulations of TECs and to provide functional information of the detected gene expression profiles. Lately, a single cell atlas of human NSCLC samples including NEC and TEC demonstrated the high phenotypic and functional heterogeneity of blood vascular ECs, distinguishing distinct subgroups of arterial, capillary and tip ECs (Goveia et al., 2020).

Moreover, Sievert et al. (2014) could not only show differences in the expression of surface markers when analyzing primary ECs originating from different organs, but also demonstrated a detailed analysis of phenotypic and functional differences between TECs and NECs. Interestingly, TECs were of larger size, could spread faster in a more chaotic architecture and were less able to adapt to blood flow conditions. In addition, tubular structure formed by TECs showed significantly less branching and looping compared to NECs as well as they up-regulated the stem cell marker CD34 as well as the angiogenesis promoting markers CD61 (Integrin β3) and CD105 (Endoglin) (Sievert et al., 2014).

Neo-angiogenesis within the tumor stroma is also known to be orchestrated tip cells. They are known to exhibit discrete marker genes, e.g., CXCR4, PGF, or ANGPT2 and on a functional level these signatures have been associated with EC migration, matrix remodeling and VEGF signaling. Therefore, tip cells have long been in the spotlight of traditional antiangiogenic therapies that mainly target VEGF. Stalk ECs have been shown to express genes involved in vessel maturation and integrity as well as DII4-Notch signaling (Blanco and Gerhardt, 2013; Goveia et al., 2020).

### TECs Are Highly Proliferative, Exhibit Abnormal Gene Expression Profiles Suggesting a Distinct Stem-Like Origin

Next to describing vessel structures and phenotypes, the characterization of possible TEC-specific genetic signatures is of particular interest. Growing evidence from both human and murine experiments show that ECs of malignant tumors prominently differ from NECs, exhibiting abnormal gene expression profiles and chromosomal instability (Akino et al., 2009; Schaaf et al., 2018). For instance, TECs display cytogenetic abnormalities such as larger nuclei and aneuploidic karyotypes as well as karyotypic abnormalities including deletions, translocations and abnormal centrosomes (Hida et al., 2004). Moreover, epigenetic alterations of TECs have been proposed. In prostate cancer Deeb et al. (2011) showed a different methylation of the gene promoter CYP24A1 in the endothelium of prostate cancer and the surrounding benign tissue, indicating an epigenetic influence on the gene expression profile of TECs. Also, Luo et al. (2013) found marked differences in the transcriptome and methylome of ECs in malignant and benign prostate tissue. Furthermore, TECs are highly proliferative in cell culture, suggesting a lack of normal mitosis inhibiting cell cycle proteins (Hida et al., 2004). All these aberrations may be rooted in the aggressive composition of the TME as it seems evident that hypoxia and reactive oxygen species (ROS) induce genetic and chromosomal instability *via* a higher mutational frequency (Kondoh et al., 2013; Hojo et al., 2017). On the other hand, pro-angiogenic factors such as VEGF can induce genetic reprogramming of TECs and their mode of interacting with immune cells (Albini et al., 2018; Maishi et al., 2019). In particular, up-regulation of angiogenic receptors as well as the close interaction with tumor cells and pro-inflammatory immune cells results in an inflamed and activated TEC state, inducing a highly proliferative phenotype with increased tendency for migration (Matsuda et al., 2010; Ohga et al., 2012).

Moreover, we want to discuss the diverse origins of TECs as a series of recent publications by Lambrechts et al. (2018); Goveia et al. (2020), and Rohlenova et al. (2020) using scRNAseq contributed to elucidate the heterogeneous origin of TECs and their corresponding untransformed EC types. These studies demonstrated that especially those NECs/TECs with capillary and venous genetic phenotype are relevant in active immune surveillance and distinguished the genetically differing subgroups of postcapillary venous ECs and activated postcapillary ECs. The postcapillary EC subgroup was mainly found within NECs and showed gene expression involved in leukocyte recruitment and tissue perfusion. Contrarily, the activated postcapillary vein EC phenotype was almost exclusively detected within the TEC population and exhibited up-regulation of immunomodulatory genes and ribosomal proteins, which have previously been described as characteristic of high endothelial venules (HEV) in inflammatory tissues. Intriguingly, VEGF blockade increased the presence of activated postcapillary venular ECs and was associated with molecular vessel normalization. Another study showed that VEGF could suppress leukocyte migration by inhibiting EC activation (*via* interference with NF-κB pathway components) or by modulating EC expression of several immunomodulatory genes, such as T cell attracting-chemokines (CXCL10 and CXCL11) (Huang et al., 2015). These data underline the immunomodulatory functions of VEGF and that targeting the VEGF-R/VEGF pathway not only impedes angiogenesis but also increases EC activation and function. Recently, by using a scRNAseq based dataset Ma et al. distinguished intra-tumoral transcriptomic heterogeneity as an unfavorable prognostic factor in primary liver cancer. In the context of tumor angiogenesis they found that VEGF is able to adversely reprogram the TME and that T cells from more heterogenous tumors had lower cytolytic activities (Ma et al., 2019).

Furthermore, it is controversially discussed if stem cell-like ECs, also called endothelial progenitor cells [EPCs, characterized by Asahara et al. (1997)] as physiological origin of ECs, contribute to tumor-vasculogenesis as well. On the other hand, there are hints that cancer cells themselves can also *trans*-differentiate into endothelial-like cells and thereby generate “their own” vessel structures. Strikingly, it was found in glioblastoma and lymphoma that TECs and cancer cells share common cytogenetic and genomic alterations (Streubel et al., 2004; Wang et al., 2010). It has further been reported that cancer cells can form pericytes (Cheng et al., 2013) and that other cell types of the TME, such as dendritic cells (DCs) and monocytes, possess the ability to differentiate into ECs (Fernandez Pujol et al., 2001; Chen et al., 2009). Moreover, TECs are able to up-regulate stem cell-associated proteins like CD90, Sca-1, MDR1 ALP, Oct-4 and high ALDH acitivity (Ohga et al., 2012; Ohmura-Kakutani et al., 2014; Hida et al., 2017) and to form stem cell-like clusters allowing increased self-renewal and high proliferative potential, which has already been described for normal ECs (Matsuda et al., 2010; Ohga et al., 2012). Importantly, TECs of highly metastatic tumors exhibit greater genetic heterogeneity (Ohga et al., 2012; Ma et al., 2019).

Hypoxia-induced signaling cascades do not only contribute to the specific TEC phenotype such as irregular cell surfaces, fenestrated cell walls and loose intercellular junctions promoting leakiness (Lin et al., 2012; Morikawa et al., 2002), but also take influence on many cellular functions of TECs, thus playing an important role in tumor development and progression. Firstly, it has been shown that ROS directly influence cellular and chromosomal instability (Kondoh et al., 2013) and that a hypoxic microenvironment is a key driver of the morphological and molecular profile of TECs (Albini et al., 2018; Maishi et al., 2019). For instance, single cell transcriptomic analyses of murine TECs revealed profoundly altered gene expression profiles under the influence of anti-angiogenic treatment, which again underlines endothelial heterogeneity (Zhao et al., 2018). Moreover, a hypoxic TME markedly enhances inflammation in TECs. Interestingly, Tellier et al. (2015) showed both *in vitro* and *in vivo* that ECs exposed to hypoxia expressed tumor-promoting pro-inflammatory cytokines such as IL-6, IL-8, CXCL1, and increased ICAM1. Likewise, hypoxia deeply influences immune responses as a hypoxic microenvironment promotes immune tolerance and angiogenesis *via* promotion of regulatory T cells (Treg) (Facciabene et al., 2011) as well as by repressing T effector cell functions (Tian et al., 2017). Furthermore, the distinct stem-like origin of TECs also depends on hypoxia. For example, Rocha et al. (2018) demonstrated that pharmacological blockade of hypoxia-associated changes prevents the differentiation of glioblastoma stem-like cells into TECs. Importantly, a hypoxic TME also impacts the regulation of angiogenesis. In this regard it has been shown that angiogenetic feedback loops by soluble VEGFR are compromised in colon cancer-derived TECs, indicating a pro-angiogenetic disposition of TECs (Jayasinghe et al., 2009). Lastly, a hypoxic micro-milieu affects protein glycosylation and may therefore impact immune cell migration to the tumor and signaling cascades within the TME (Chandler et al., 2019).

These different origins and stem cell populations of TECs and the impact of hypoxia on these cell populations may at least in part explain their striking heterogeneity and are associated with drug resistance and worse clinical outcome (Naito et al., 2016; Hida et al., 2017). Thus, TECs are certainly not normal EC, but are yet themselves abnormal in terms of their cytogenetics, genetic expression profile, methylation patterns and their functional behavior. Thus, TECs play a major role in driving the TME to be highly tumor-permissive.

## TECs and Their Immunoregulatory Properties

**FIGURE 1:**
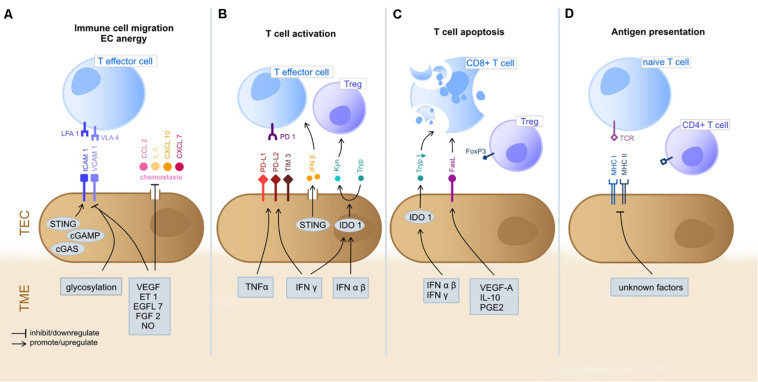
Immunoregulatory functions of TEC in the TME. The gray boxes indicate TME-derived factors that inhibit/promote expression of mediators by TEC. **(A)** T cell extravasation into the TME starts with a multi-staged adhesion process and includes binding of integrins LFA1 and VLA4 on T cells to the respective ligands ICAM1 and VCAM1 on TEC (Ley et al., 2007; Georganaki et al., 2018). TEC can actively downregulate gene expression of adhesion molecules (e.g., ICAM1 and VCAM1) or chemostaxis themselves in order to control immune cell infiltration (Griffioen et al., 1996; Lambrechts et al., 2018). TME deriving cytokines (e.g., VEGF, ET1, EGFL7, and FGF2) inhibit TEC to upregulate expression of adhesion molecules and chemoattractants (e.g., CCL2, IL6, CXCL10, and CXCL7) (Flati et al., 2006; Buckanovich et al., 2008; Delfortrie et al., 2011). Also, NO has been shown to inhibit adhesion molecule and cytokine (IL6, IL8; not shown) expression (De Caterina et al., 1995). The intracellular cGAS-cGAMP-STING pathway is known to enhance adhesion molecule and promote T cell infiltration (Demaria et al., 2015). The glycosylation of surface molecules (referred to as glycocalyx) modulates the adhesive properties of TEC and can either enhance or reduce immune cell migration (Chandler et al., 2019). **(B)** T cell activation starts at the TEC/TME interface. Binding of inhibitory immune checkpoints (e.g., PD-1) on CD8+ cells with their ligands (e.g., PD-L1 and PD-L2) on TEC inhibits T cell activation and these ligands can be upregulated by TEC on proinflammatory TME derived factors (IFNγ and TNFα) (Georganaki et al., 2018). STING (see above) acts T cell activating via IFNβ secretion (Demaria et al., 2015). The immunosuppressive cytosolic protein IDO1 promotes the metabolism of Tryp to Kyn. Kyn downstream metabolites promote Treg activation, whilst Tryp depletion promotes T cell apoptosis and inhibits T cell proliferation. TME derived type I IFN (alpha/beta) upregulate IDO1 expression by TEC (Munn and Mellor, 2016; Georganaki et al., 2020). **(C)** T cell apoptosis can be triggered by IDO1 depended depletion of Tryp (Georganaki et al., 2020). Moreover, the molecule FasL expressed by TEC promotes CD8+ T cell apoptosis whilst sparing Treg (due to expression of FoxP3) and is FasL expression is upregulated in response to TME derived VEGF-A, IL-10 and PGE2 (Motz et al., 2014). TEC, tumor endothelial cells; TME, tumor microenvironment; LFA1, lymphocyte function-associated antigen 1; VLA4, very late antigen-4; ICAM1, Intercellular Adhesion Molecule 1; VCAM1, Vascular cell adhesion protein 1; VEGF, Vascular Endothelial Growth Factor; ET-1, Endothelin-1; EGF-like domain-containing protein 7; FGF2, Fibroblast Growth Factor 2; NO, nitric oxide; PD-1, programd death receptor 1; PD-L1/2, programed death receptor ligand 1/2; Tryp, tryptophan; Kyn, kynurenine; IFN, interferon; IDO1, indoleamine 2,3-dioxygenase 1; MHC, major histocompatibility complex; FasL, Fas ligand. **(D)** TEC can present processed antigens to T cells via MHC I and MHC II molecules, yet they are lacking the co-stimulatory markers CD80 and CD86 required for naïve T cell activation, distinguishing them from professional APC (Kambayashi and Laufer, 2014). Contrarily, CD4+ T cells are not dependent on co-stimulation at may directly be activated by TEC (Shiao et al., 2007). TEC can actively downregulate HLA genes as immune evading strategy (Goveia et al., 2020), yet external TME-derived factors have not been specified yet.

Tumor endothelial cells are strategically positioned at the TME-vessel interface and represent the first-line encounter for circulating immune cells and the tumor stroma (Lambrechts et al., 2018). TECs are known to impact TME immunogenicity by actively guiding adhesion, rolling and extravasation of circulating immune cells into the tumor stroma, but also by fulfilling immune regulatory properties themselves, such as T cell priming and activation as well as exerting antigen presenting functions (Kambayashi and Laufer, 2014; Georganaki et al., 2018). The functional immunomodulatory characteristics will be discussed in the following (Figure 1).

### Immune Cell Transmigration: Attraction, Adhesion, and Perfusion

In healthy as in cancerous sites, the process of immune cell extravasation and tissue infiltration involves a complex process of multi-staged adhesion and transcellular migration and is greatly mediated by activated venous ECs.

The leukocyte adhesion cascade includes the three main steps of selectin-mediated rolling, chemokine-mediated activation (e.g., CXCL4) and integrin-mediated arrest of leukocytes. ECs play a major role in leucokyte adhesion by expressing selectins (e.g., E-selectin and P-selectin), integrin ligands (e.g., VCAM1 and ICAM1) and adhesion molecules (e.g., glycosaminoglycans). Following adhesion, leukocyte transendothelial migration may be paracellular or transcellular. The more frequent paracellular form of migration involves the adhesion molecules PECAM1 (also known as CD31) and CD99. Transcellular migration mainly takes place in areas of thin endothelial linings (e.g., central nervous system) and involves vesiculo-vacuolar organelles (VVO) that form intracellular channels for leucokyte migration. As a last step, leukocytes pass the endothelial basement membrane and pericyte sheath, mostly in areas of low protein deposition and through pericyte gaps (Ley et al., 2007). Importantly, only activated ECs can initiate leukocyte migration and EC activation itself greatly relies upon stimulation by TNFα, mainly *via* activation of the NF-κB pathway (Huang et al., 2015). Quiescent ECs do generally not interfere with leukocytes by suppressing transcription of adhesion molecules (e.g., ICAM1, VCAM1, and E-selectin) (Pober and Sessa, 2007).

### Tumor Endothelial Anergy

Inhibiting leukocyte migration into the TME at the endothelial level depicts one major way of cancer cells to escape anti-tumor host immune responses. Endothelial anergy describes the unresponsiveness of TECs to pro-inflammatory stimulation (TNFα, IFNγ, and IL-1^10^), which greatly impedes the adhesion and migration of immune cells. Accompanying the anergetic state, TECs can actively downregulate the expression of adhesion molecules (e.g., ICAM1, VCAM1, and E-selectin) or chemokines (e.g., CCL2, CCL18, CXCL10, and CXCL11) themselves (Griffioen et al., 1996; Lambrechts et al., 2018), thus reducing immune cell trafficking. Moreover, TECs select specific subsets of immune cells to infiltrate the tumor tissue, favoring Treg and macrophage precursors (MDSC), but inhibiting CD4^+^ and CD8^+^ effector T cells, DCs, natural killer (NK) cells and neutrophil granulocytes (De Sanctis et al., 2018). This consequently promotes an immunosuppressive tumor milieu, ultimately giving rise to tumor progression (Zhang et al., 2003; Bouma-ter Steege et al., 2004).

Tumors can upregulate several cytokines and chemokines in order to promote endothelial anergy. Most prominent, members of the VEGF family and fibroblast growth factor 2 (FGF2) inhibit the upregulation of cell adhesion molecules on TECs (e.g., ICAM1, VCAM1, and selectins) under inflammatory stimulation (e.g., TNFα) (Griffioen et al., 1999; Dirkx et al., 2003; Flati et al., 2006). Additionally, EGF-like domain-containing protein 7 (EGFL7) downregulates adhesion molecule expression and directly fosters tumor vessel development *via* NOTCH signaling (Delfortrie et al., 2011; Nichol and Stuhlmann, 2012). Recently it has been shown that nitric oxide (NO), a known physiological factor of endothelial relaxation, seems to essentially regulate blood flow within tumors (Fukumura et al., 2006). NO also affects leukocyte recruitment by preventing rolling and adhesion of immune cells under malignant conditions (De Caterina et al., 1995) and has been reported to directly suppress effector T cells (De Sanctis et al., 2018). The vasoconstrictive peptide Endothelin 1 (ET1), known for its direct effect on angiogenesis *via* VEGF and HIF1 (Spinella et al., 2002), is also associated with ICAM1 expression and the decreased presence of tumor infiltrating leukocytes (TIL) (Buckanovich et al., 2008). Another mechanism of endothelial anergy includes the secretion of soluble adhesion molecules by tumor cells (MCAM/sCD146 and Endoglin) that indirectly inhibit TIL recruitment by competing with EC bound-receptors (Rossi et al., 2013; Stalin et al., 2016) and by direct VEGF-synergistic interactions with tumor angiogenesis (Zheng et al., 2009). Furthermore, TECs can upregulate the expression of specific adhesion markers in order to direct immune cells. In this regard the common lymphatic endothelial and vascular endothelial receptor (CLEVER 1) has been identified as immunosuppressive molecule, favoring the influx of Treg as well as tumor associated macrophages (TAM) (Nummer et al., 2007; Karikoski et al., 2014).

Of note, endothelial anergy is reversible and may be used as a therapeutic strategy. Pre-clinical data suggests that blocking the before mentioned mechanisms in TECs favors the influx of immune cells (Dirkx et al., 2006; Karikoski et al., 2014; Facciabene et al., 2017). Reports from clinical data suggest that endothelial anergy is associated with inferior outcome (Takahashi et al., 2001; Wu et al., 2009; Huang et al., 2010) and that anti-VEGF therapies may contribute to reprogramming of the TME (Griffioen et al., 1999; Huang et al., 2015). The immune modulatory effects of anti-angiogenic therapies and immunotherapies such as immune checkpoint inhibitors (ICI) thus applies a dual targeting concept, allowing a more effective reinvigoration of the anti-cancer immune response. Endothelial cell anergy complements the concept of immune cell anergy, which is defined as the failure of immune cells to create an effective response against cancer cells (Thommen and Schumacher, 2018). Targeting both mechanisms thus may be additive or even synergistic in case the most appropriate targets are well defined.

### TEC Affects T Cell Priming and Migration

Next to altering leukocyte extravasation, TECs can actively impact T lymphocyte priming and migration (Figures 1A,B). This fact is especially of clinical interest, as many novel therapeutic strategies include immune checkpoint inhibitors (ICI) up-front and thereby target effector T cells in the TME.

The role of TECs in T cell activation is two-sided as both stimulatory and inhibitory mechanisms have been identified. As for the latter, TECs can up-regulate a variety of inhibitory molecules in order to promote T cell arrest. One important mechanism involves immune checkpoint molecules (IC) that get expressed by various cells of the TME (e.g., cancer cells, immune cells, and stromal cells). The most (clinically) important immunoinhibitory IC involves programmed death receptor (PD-1) which is found on CD8^+^ T cell surfaces. Binding of PD-1 with its ligands (PD-L1 and PD-L2) down-regulates T cell activation. Next to cancer cells, TECs have been shown to express PD-L1 and PD-L2 themselves, as well as other known inhibitory ICs, such as T-cell immunoglobulin domain and mucin domain (TIM-3), thereby having the potential to directly inhibit T cell activation at the vessel site. It has been shown that pro-inflammatory cytokines including IFNγ and TNFα enhance PD-L1 up-regulation on ECs. PD-L1 expression by cancer cells is currently the most frequently used biomarker in immune-oncology guiding treatment decisions and patient stratification, However, the therapeutic impact of PD-L1 expression by TECs has not yet been specified (Georganaki et al., 2018).

Another T cell inhibitory mechanism of TECs involves Fas ligand (FasL), a homeostatic mediator of T cell apoptosis. An interesting preclinical study found that TECs expressing FasL were able to effectively diminish CD8^+^ T cells in tumors while sparing Treg cells. FasL expression was induced by tumor-derived VEGF-A, IL-10 and prostaglandin E2 and pharmacological inhibition of these factors resulted in lower FasL expression and consequently higher CD8^+^ T cell infiltration by preventing effector T cell apoptosis (Motz et al., 2014) (Figure 1C).

Furthermore, the enzyme IDO1 is known for its immunosuppressive functions by restricting T cell proliferation and inducing T cell apoptosis as well as promoting Treg activation *via* its key role in tryptophan metabolism. Next to other cells of the TME (e.g., cancer and immune cells), TECs can express and up-regulate IDO1 in response to type I IFN (αβ) and type II IFN (γ) stimulation in the TME, which has been associated with impeded T cell activation, thus promoting an immunosuppressive microenvironment. IDO inhibition has been shown to enhance efficacy of ICI and trials to establish the clinical use are ongoing (Georganaki et al., 2020).

As to T cell promoting mechanisms, the intracellular STING (the stimulator of IFN genes) signaling represents an important innate immune pathway in healthy and cancerous tissues also promoting CD8^+^ T cell activation *via* expression of type I IFN (especially IFN-β). STING gets ubiquitously expressed by various cells of the TME, and especially high expression has been documented for ECs of high endothelial venules (HEV). STING activation leads to CD8^+^ T cell tumor infiltration and has also been shown to enhance the upregulation of adhesion molecules (e.g., ICAM1 and VCAM1) on ECs, alterations that altogether promote anti-cancer immune responses. Moreover, there is evidence that STING activation is involved in tumor vessel maturation and inhibition of vessel sprouting. Furthermore, it could be shown that the cGAMP-induced antitumor activity was found to be mediated by the STING-driven induction of IFN-β in the TME. Interestingly, TECs were the main producer of IFN-β in response to cGAMP injection in both mouse and human (Demaria et al., 2015). Therefore, STING agonists are interesting modulators of the TME and not only affecting immune cells, as mostly appreciated, and therapeutic development is already ongoing in the clinical setting (Yang et al., 2019).

Next to TECs, LECs of extra-tumoral lymph nodes are known to chemo-attract and cross-prime naïve CD8^+^ T cells by acting as semi-professional APCs (see below). Recently, LECs have been shown to generate antigen-experienced T cells with memory-like function that can rapidly evolve effector functions upon pro-inflammatory stimulation. These results give first insights into the functional consequence of LEC-associated T cell priming (Vokali et al., 2020).

### TEC as Antigen Presenting Cells

It is well established that ECs from non-cancerous vasculature can express MHC class I and II molecules enabling them to present processed antigens to T cells (Figure 1D). Yet, there is currently no evidence that ECs can express the co-stimulatory markers CD80 and CD86 required for naïve T cell activation (Kambayashi and Laufer, 2014), distinguishing them from professional APCs. It seems they can only stimulate antigen experienced T cells. As conversely shown in a xenograft-model, CD4^+^ memory cell response is not strictly depended on co-stimulation but can directly react on EC stimulation (Shiao et al., 2007). This suggests a contributing role of ECs in CD4^+^ T cells tolerance. *Vice versa* CD4^+^ T cells have been identified to mediate vessel normalization processes by directly altering associated gene expression of TECs (e.g., decrease of adhesion and ECM molecules, increase of VEGFA) (Tian et al., 2017).

Interestingly, liver sinusoidal ECs bear the capacity to cross-present antigens to naïve T cells, causing their rapid differentiation into memory T cells. Although these were not TECs, this is another line of evidence for ECs as a T cell tuning cell type (Knolle and Wohlleber, 2016).

It was recently shown in single cell analysis of TEC and NEC that especially those ECs with capillary phenotype harbor genes associated with MHC II-mediated antigen presentation. Moreover, TECs can downregulate genes responsible for MHC expression (HLA-DQB1 and HLA-DQA1) impeding their antigen presenting functions, thus contributing to tumor immune-evasion (Goveia et al., 2020). The exact role of ECs, and even more of TECs as APCs remains to be specified in future studies, however, one potential mechanism could be the cross presentation of antigens.

## High Endothelial Venules (HEVs) and Vascular Growth Factors Are Involved in the Formation of Tertiary Lymphoid Structures (TLS)

Tertiary lymphoid structures (TLS) have very recently been identified as an important prognosticator in many different tumor types such as melanoma, colorectal, pancreatic and lung cancer (Martinet et al., 2012; Goc et al., 2014; Hiraoka et al., 2015; Posch et al., 2018; Cabrita et al., 2020; Helmink et al., 2020). Moreover, TLS harbor a response-predictive potential, having been associated with enhanced response to ICI (Cabrita et al., 2020; Helmink et al., 2020).

TLS are defined as a neoformation of lymphoid tissues in peripheral inflammatory environments such as organ transplants, infectious, autoimmune and inflammatory diseases as well as in tumors (Sautès-Fridman et al., 2019). TLS share many characteristics with physiological secondary lymphoid organs (SLO) such as lymph nodes, which represent the main interface between peripherally matured DCs and CD4^+^ and CD8^+^ T cells (Mellman et al., 2011). This interaction instructs effector T cells (T_*eff*_) to migrate to the tumor site and convey anti-cancer immune response (Mellman et al., 2011; Sautès-Fridman et al., 2019). Under malignant conditions, TLS similarly represent a crucial site for the interaction of tumor antigen-presenting DCs and T cells as well as for the proliferation of B and T cell subpopulations (Germain et al., 2014; Goc et al., 2014; Kroeger et al., 2016). Thus, it can be expected that the presence of TLS eases immune cell recruitment and instructs tumor immunity (Sautès-Fridman et al., 2019). Recent publications using multi-omic approaches (Cabrita et al., 2020; Helmink et al., 2020) described the coincident presence of CD20^+^ tumor infiltrating B cells and tumor associated CD8^+^ T_*eff*_ cells, particularly during ICI, suggesting that these B cells may eventually contribute to T cell instruction by functioning as APCs.

**FIGURE 2:**
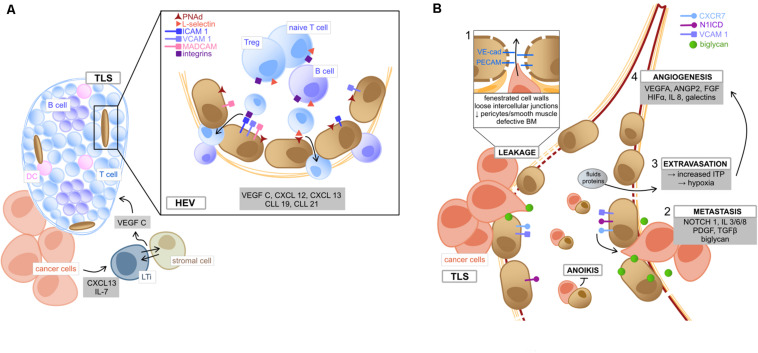
Tumor endothelial cells **(A)** of high endothelial venules and **(B)** their role in metastasis. **(A)** Within the TME, tumor secreted cytokines (CXCL13 and IL7) induce the formation of LTi, which tightly interact with adjacent stromal cells to produce VEGF C, lastly inducing the formation of TLS (Furtado et al., 2007; Meier et al., 2007; Colbeck et al., 2017a). HEV are located in the TLS periphery and mediate immune cell transmigration. TEC lining HEV are characterized by the HEV specific marker PNAd. Binding of T cell expressed L-selectin to PNAd on TEC implies the first step of lymphocyte adhesion, followed by further interactions, such as binding of integrin ligands (ICAM1, VCAM1, and MADCAM1) with the respective receptors (LFA1, VLA4, and α4β7) (Jones et al., 2018). Lymphocytes can then transmigrate into the structured zones of the TLS, where they become activated upon interaction with local DC (Sautès-Fridman et al., 2019). TME deriving chemokines and angiocrine factors (gray box) mediate the migrating process as well as TLS organization (Sautès-Fridman et al., 2019). **(B)** The metastatic process in the TME is fired by the leaky and weak architecture of tumor vessels (box 1, involving loose connection of intercellular adhesion molecules VE-cad and PECAM), which facilitates cancer cells to disintegrate from the surrounding matrix and migrate into tumor vessels via binding to the chemokine receptor CXCR7 (Maishi et al., 2019). This metastatic process (box 2) is fostered by TME deriving pro-inflammatory cytokines (e.g., IL 3/6/8) and growth factors (e.g., PDGF and TGFβ) (Maishi et al., 2019). Moreover, NOTCH 1 signaling in TEC as well as expression of activated NOTCH 1 receptors (N1ICD) facilitate metastasis, e.g., via upregulation of integrin ligand VCAM1 (Wieland et al., 2017). Also, the TEC-expressed angiogenic molecule biglycan promotes hematogenous cancer cell metastasis (Maishi et al., 2016). Circulating TEC can bind to metastasizing cancer cells and exhibit protection from apoptosis (anoikis) (Cao et al., 2016). Besides, vessel leakiness leads to extravasation of fluids and proteins, which increases ITP, resulting in hypoxia and (box 3), the release of angiogenic factors by cellular TME components (box 4) and consequently promotes tumor neo-angiogenesis (Butler et al., 2010). TLS, tertiary lymphoid structures; TME, tumor microenvironment; LTi, lymphoid tissue inducer cells; HEV, high endothelial venules; PNAd, peripheral lymph node addressin; ICAM1, intercellular adhesion molecule 1; VCAM1, vascular cell adhesion protein 1; MADCAM1, mucosal addressin cell adhesion molecule-1; LFA1, lymphocyte function-associated antigen 1; VLA4, very late antigen-4; VE-cad, vascular endothelial cadherin; PECAM, platelet endothelial cell adhesion molecule; PDGF, platelet derived growth factor; TGFβ, transforming growth factor beta; N1ICD, NOTCH1 intracellular domain; ITP, intra-tumoral interstitial pressure.

Similar to SLO, TLS are closely associated with specialized vessels called high endothelial venules (HEV), which are located in the peripheral zone of the TLS (Martinet et al., 2011) and characteristically express PNAd, MECA79 and selectin ligands (Jones et al., 2018), all of which are required for lymphocyte homing (Streeter et al., 1988; Girard and Springer, 1995) (Figure 2A). Tumor-secreted cytokines (CXCL13 and IL-7) induce the formation of lymphoid tissue inducer (LTi) cells (Meier et al., 2007), which interact with local stromal cells via Lymphotoxin-α1β2 (Colbeck et al., 2017a). Consequently, these cells release the angiogenic factor VEGF-C, which is essential for HEV formation (Furtado et al., 2007). Like under other circumstances, EC under the stimulus of various chemokines (CXCL12, CXCL13, CCL19, and CCL21) mediate the recruitment of central cell populations of anti-tumor immunity by the expression of surface markers (Luther et al., 2002; Fleige et al., 2014; Sautès-Fridman et al., 2019). HEV have been reported to express ICAMs, VCAMs, and MADCAMs in a great density fostering the recruitment of lymphocyte population and organize them into zones (Girard and Springer, 1995). Furthermore, the ECs of lymphatic vessels associated with TLS express Podoplanin and secrete CCL21, which favors emigration of educated lymphocytes to the draining lymph nodes (Ruddle, 2016). This fact could consequently promote recognition of tumor antigens and contribute to establish a sustained protective immunity. Therefore, the EC in HEV possess an important gatekeeper function in TLS-associated immune response.

The selective influx of lymphocyte populations by regulatory EC seems to be of particular importance in the context of immunotherapy. As an example, Treg depletion (known to induce immune-mediated cancer regression) is linked to HEV development and consequently increased formation of TLS (Hindley et al., 2012; Colbeck et al., 2017b). From a clinical perspective, there is evidence that the presence of HEV acts as a favorable prognostic factor in melanoma (Martinet et al., 2012), pancreatic cancer, NSCLC and colorectal cancer (CRC) (Goc et al., 2014; Hiraoka et al., 2015; Posch et al., 2018). Moreover, TLS density depicts a predictive factor of response to anti-PD-1/PD-L1 antibodies. The putative mechanisms remain poorly understood, but could at least in part be rooted in the enhanced functionality of tumor infiltrating B lymphocytes (Cabrita et al., 2020; Helmink et al., 2020). Interestingly, the combination of anti-PD-L1 therapy and anti-angiogenic therapies further increases HEV and subsequent TLS formation (Allen et al., 2017). The mechanisms behind the synergistic effect of dual VEGF- and PD-1/PD-L1-targeting therapies remain incompletely understood, yet the concept of transforming cold immune-suppressed tumors into hot immune-competent tumors is appealing (Sautès-Fridman et al., 2019). The concept is currently mainly based on pre-clinical observations, highlighting the potential of combined targeting of VEGFR2, PDL1 and LTβ in enhancing HEV and TLS formation and, consequently, in tumor destruction (Allen et al., 2017; Colbeck et al., 2017b). Thus, it has been formulated very recently that the induction of intratumor HEV and TLS represents an important future target to improve anti-cancer immunity (Sautès-Fridman et al., 2019).

## The Role of Tumor Lymphoid Vessels Harboring Endothelial Cells With VEGF-C/VEGFR3 Signaling Activity in the Occurrence of Lymphoid Metastases

Endothelial cells not only coat blood vessels, but also form the inner lining of lymphatic vessels, which are usually dependent on VEGF-C and VEGFR3 signaling (Karkkainen et al., 2004). There is emerging insight that VEGFR3 positive ECs play an important (patho)physiological role not only in lymphangiogenesis of tumors as the structural determinant for lymph-node metastasis, but also in chronic inflammatory diseases (Hsu et al., 2019). The VEGF-C/VEGFR3 axis was found to be up-regulated in various tumor entities such as genitourinary tract, gastrointestinal and prostate cancer (Yu et al., 2013; Wang et al., 2014; Yang et al., 2014; Zhu et al., 2016). Pre-clinical and clinical evidence demonstrated that VEGF-C fosters the formation of lymph vessels by promoting lymphatic outgrowth, ultimately enabling lymphatic metastasis (Skobe et al., 2001; Mattila et al., 2002; Paiva et al., 2015; Zhu et al., 2016; Yeh et al., 2017). Consequently, the expression of VEGFR3 was correlated with lymph node and distant metastasis and poor prognosis in many different cancer types (Decio et al., 2014; Wang et al., 2014; Yang et al., 2014; Goussia et al., 2018). As shown in ovarian and breast cancer, tumor cell expression of VEGFR3 may generate a proliferative advantage by autocrine and paracrine signaling (Decio et al., 2014; Varney and Singh, 2015).

The VEGF-C/VEGFR3 axis may also be involved in anti-cancer immune responses. As shown in an interesting murine melanoma model, VEGF-C activated LECs exhibit immunomodulatory functions by suppressing tumor-specific CD8^+^ T cells by lymphatic antigen presentation (Lund et al., 2012). Moreover, NK cells isolated from patients with acute myeloid leukemia (AML) were found to express increased levels of VEGFR3 and lower levels of IFN-γ (Lee et al., 2013). Importantly, resident macrophages in lung adenocarcinoma also expressed VEGFR3 and pharmacological inhibition of VEGFR3 signaling sensitized to chemotherapy (Li et al., 2017).

Several FDA-approved drugs target VEGF-C/VEGFR3, such as sorafenib, sunitinib pazopanib, axitinib (Hsu et al., 2019). For patients with renal cell carcinoma (RCC), hepatocellular carcinoma (HCC), thyroid cancer, gastrointestinal stromal tumors (GIST) and soft tissue sarcoma treated with these TKIs improved outcomes were reported (Demetri et al., 2006; Escudier et al., 2007; Motzer et al., 2007, 2017; Llovet et al., 2008; Cheng et al., 2009; Sleijfer et al., 2009; Sternberg et al., 2010; Brose et al., 2014; Haas et al., 2016; Ravaud et al., 2016). Accordingly, the monoclonal antibodies bevaczizumab and ramucirumab targeting VEGF and VEGF signaling improved outcomes for patients with NSCLC, CRC and gastric cancer (Hurwitz et al., 2004; Sandler et al., 2006; Miller et al., 2007; Ohtsu et al., 2011; Garon et al., 2014; Tabernero et al., 2015). Conceivably, off-target effects on VEGF-C/VEGFR3 of these drugs may at least in part contribute to their anti-cancer efficacy, even though this has not formally been proven in the clinical setting.

## TECs and Their Signaling Properties (Angiocrine Factors) Modulate the TME and Foster Tumor Progression and Metastasis as Well as Therapy Resistance

Cancer progression and metastasis is a highly complex and multifactorial process depending on many factors and different cell types. Even though CAFs and suppressive immune cells very much contribute to cancer cell progression and metastatic spread (Quail and Joyce, 2013; Maishi et al., 2019), we will here focus on TEC (Figure 2B). Firstly, the abnormal and leaky architecture of tumor vessels facilitates the disengagement of tumor cells and the hematogenous metastatic spread of cancer cells (Maishi et al., 2019) (Figure 2B). Mechanistically, the adhesion molecule expression profile of TEC functions as scaffold guiding malignant cells to intravasation (Sökeland and Schumacher, 2019). Moreover, TEC were shown to use metalloproteases to break through the vessel basement membrane, thus directly impacting metastasis (Ohga et al., 2012).

Furthermore, angiocrine factors centrally contribute to cancer progression and metastasis (Butler et al., 2010), including numerous growth factors (bFGF, G/GM-CSF, IGF1, PDGF, and TGFβ), interleukins (IL-3, 6, 8) and other factors like NOTCH, calcineurin, biglycan, Jag1, and Slit2 (Maishi et al., 2019). Several mechanisms for the initiation of metastasis by angiocrine factors have been described. For instance, Notch1 signaling in TEC was observed to up-regulate chemokines and adhesion molecules (VCAM1) inducing leukocyte adhesion and metastatic outgrowth (Wieland et al., 2017). Moreover, the chemokine receptor CXCR7 initiates and mediates transendothelial migration of tumor cells under the control of TEC. In addition, angiocrine factors facilitate the blocking of tumor suppressive signals. For instance, the tumor suppressive factor Slit2 can directly be downregulated by TEC *via* their receptor EphA2 in a paracrine manner (Brantley-Sieders et al., 2011). However, TEC may also have an influence on therapy resistance *via* these angiocrine factors (Maishi et al., 2019). As an example, direct interaction of TEC and lymphoma cells *via* FGF4/FGFR1 has been shown. TEC express Jag1, which helps to generate a malignant vascular niche that correlates with aggressive course and resistance to chemotherapy (Cao et al., 2014).

Anoikis is defined as apoptosis of cells that have lost their contact to the extracellular matrix (ECM) (Frisch, 1994) and may play a crucial role in the formation of metastasis (Cao et al., 2016) (Figure 2B). EC use their surface molecules to connect with cell matrix contacts and it is thus speculated that circulating TEC attach to tumor cells and prevent anoikis-mediated apoptosis (Yadav et al., 2015).

Biglycan is a small leucin-rich repeat proteoglycan functioning as an angiocrine factor and is expressed by TEC of highly metastatic tumors (Maishi et al., 2016). It is suggested that biglycan allows toll-like receptor expressing tumor cells to metastasize hematogenously. Interestingly, biglycans seem to be also subject to epigenetic regulations (Maishi et al., 2016).

The glycosylation of surface proteins of cell populations in the TME seems to be another regulatory factor and mechanism of tumor progression and metastasis. EC exhibit a great extent of glycosylated proteins, which can be influenced by inflammation and hypoxia (Chandler et al., 2019). Glycosylation of endothelial adhesion molecules (e.g., ICAM1, VCAM1, PECAM, and lectins) affects the cellular adhesive properties of TEC. Thus, tumor infiltration by immune cells can either be favored or prevented (Chandler et al., 2019). Moreover, the signaling, adhesion and migration of EC can be dysregulated by altered glycosylation. Accordingly, VEGFR2 signaling can be negatively influenced and formation of abnormal vessels with an increased degree of leakage is favored (Chandler et al., 2019) finally impacting tumor progression and metastasis (Oliveira-Ferrer et al., 2017).

Galectins are a group of glycan-binding endogenous lectin proteins, which show interactions with fibroblasts, endothelial and immune cells. There is growing evidence that several isoforms of this protein family are involved in the function and regulation of the TME (Elola et al., 2018). Accordingly, it has been observed that dysregulated expression of galectins in human tumors is associated with greater extent of vascularization and unfavorable course such as rapid progression and metastatic disease (Liu and Rabinovich, 2005; Laderach et al., 2013). Here we want to focus on TEC derived from blood and lymphatic vessels. Firstly, members of the galectin family have been shown to facilitate tumor angiogenesis, as they are synthesized by activated ECs and are important mediators of EC migration and tube formation (Nangia-Makker et al., 2000; Thijssen et al., 2006; Hsieh et al., 2008; Delgado et al., 2011; Croci et al., 2014), in blood as in lymphatic vessels (Cueni and Detmar, 2009; Chen et al., 2016). Therefore, tumor angiogenesis is sustained by galectins *via* autocrine and paracrine mechanisms (Thijssen et al., 2010) as well as by direct interactions with VEGFR2 and HIF1α (Markowska et al., 2011; Zhao et al., 2011; Laderach et al., 2013; White et al., 2014). In addition, galectins seem to influence other prominent angiogenic stimuli, such as VEGF-A and bFGF (Markowska et al., 2010). Interestingly, Gal-1 activates VEGFR2 by phosphorylation resulting in unresponsiveness to anti-VEGF therapy, whereas elimination of Gal-1 restored sensitivity to anti-angiogenic therapy (Croci et al., 2014). Moreover, depletion of Gal-1 contributed to vessel normalization by enhanced pericyte coverage and increased immune recruitment (Croci et al., 2014). This also holds true for lymphangiogenesis, as Gal-8 potentiates VEGFR3/VEGF-C signaling (Chen et al., 2016). Furthermore, galectins influence the immune compartment by inducing T cell apoptosis, impairing NK function as well as favoring the proliferation of Treg, tolerogenic DC and tumor-promoting macrophages and MDSC (Elola et al., 2018).

## TECs Influence Response to Anti-Angiogenic and Immune Checkpoint Inhibitor Therapies That Contribute to Re-Regulate the TME

Throughout this review we already highlighted the immune regulatory properties of TEC and it seems obvious to discuss the additive effect and possible synergism of immunotherapy and anti-angiogenic therapies. As they mutually influence each other, reversing endothelial anergy to foster T cell infiltration and enhancing the efficacy of ICI seem promising (Uldry et al., 2017; De Sanctis et al., 2018).

The first aspect is that VEGFs are immunosuppressive, as they inhibit T cell infiltration and decrease maturation and function (antigen presentation) of DC, expand Treg and foster PD-1 expression (Ohm and Carbone, 2001; Voron et al., 2015). In addition, anti-angiogenic therapies were reported to up-regulate the expression of adhesion molecules and thereby promote the transmigration of immune cells (Griffioen et al., 1999; Dirkx et al., 2006). Thus, a better response to immunotherapy by decreasing TEC-driven immunosuppression *via* blockade of the VEGF axis is argued (De Sanctis et al., 2018). Accordingly, the formation of HEV and TLS as essential factors of anti-tumor immunity could be initiated by combining anti-VEGF and anti-PD1/PD-L1 therapies (Allen et al., 2017). Other mediators of endothelial anergy have been identified as promising therapeutic targets to restore anti-cancer immune responses (Uldry et al., 2017). For instance, NO antagonists were able to restore T cell adhesion to ECs *via* up-regulation of adhesion molecules (Bouzin et al., 2007; Buckanovich et al., 2008). Also, the stabilization of TNFα by vessel normalization lead to improved T cell function (Schmittnaegel et al., 2017). *Vice versa*, tumor angiogenesis is also driven by suppressive immune cells (MDSCs and TAM) and direct targeting of these cell populations increases sensitivity to anti-angiogenic therapy (Ugel et al., 2015a). Likewise, immunotherapy approaches were shown to induce the recognition and destruction of tumor vasculature (Ugel et al., 2015b), which was also demonstrated in a study applying CAR-T cells model targeting VEGFR2 (Chinnasamy et al., 2010; Kanagawa et al., 2013).

Vascular endothelial growth factor-targeted therapies rather reduce the tumor growth rate than inducing direct tumor shrinkage (Bagri et al., 2010) and contribute to blood flow normalization to the tumor. Consequently, the concept of vessel normalization is well established and is associated with increased sensitivity to chemo- and/or radiotherapy (Carmeliet and Jain, 2011; Uldry et al., 2017; De Sanctis et al., 2018). Retaining the TME from excessive hypoxia depicts an important factor for adequate anti-tumor immune responses (Uldry et al., 2017). For example, hypoxia induces the up-regulation of HIF1α, the increased expression of CXCL12/CXCR4 and subsequent recruitment of TAM, MDSC, and Treg (Ebos and Kerbel, 2011) as well as it favors an M2-phenotype (Facciabene et al., 2011; Colegio et al., 2014) and reduces cytotoxic T cell activity (Calcinotto et al., 2012; Barsoum et al., 2014). Anti-VEGF therapeutics therefore stabilize the tumor vasculature by vessel normalization which in turn increases anti-tumor immune responses by favoring an immune-competent TME (Huang et al., 2013; Uldry et al., 2017). To further optimize this approach, the combination of HIF1α blockers and anti-VEGF therapies further increase immune responses in pre-clinical mouse models (Chen et al., 2015) also decreasing tumor metastasis (Maes et al., 2014) via NOTCH1 mediated intravasation of cancer cells (Sonoshita et al., 2011). On the contrary, vessel normalization is also driven by the influence of immune cells, as TH1 cells are considered to crucially impact the regulation of tumor blood flow (Tian et al., 2017). Thus, vessel normalization substantially contributes to revise endothelial anergy and a mutual regulation of angiogenesis and immune response seems obvious. Combining immunotherapy strategies and blockade of the VEGF axis seems promising, changes tumor vasculature and immune responses in melanoma (Hodi et al., 2014) and has improved outcomes of NSCLC patients in clinical studies (Socinski et al., 2018).

## Conclusion

TEC are central players orchestrating the TME. They phenotypically differ from NEC, are highly proliferative, exhibit genetic instability and a stemness gene signature. Functionally, TEC are deeply involved in immune-regulation of the TME as they can exert APC-like functions and may play a role in T cell priming or T cell anergization. They are immune-regulative, as they modulate trafficking of immune cells and by favoring recruitment of immunosuppressive rather than immune effector cells, contributing to cancer immune escape. Moreover, TEC are deeply involved in the formation of TLS, which correlate with improved prognosis and better response to ICI. In addition, EC of cancer associated lymphatic vessels and their VEGF-C/VEGFR3 signaling are critical for with lymphatic metastasis. Lastly, several angiocrine factors of TEC may directly foster cancer progression and the formation of distant metastasis.

Following these key immunomodulatory characteristics of TEC, it depicts a main future challenge to further functionally characterize TEC and TEC subtypes with respect to their cancer promoting properties. Technological advances like scRNAseq have already broadened the understanding of the cellular and molecular TME composition by deducing gene sequences and defining cell sub-clusters that strongly indicate their functional dysregulation in cancer. Nevertheless, it is of great importance to sustain these findings *in vitro* and most notably *in vivo*, but the development of adequate pre-clinical models still remains challenging.

## Author Contributions

LN, LH, AP, and DW developed the concept of the review. LN and LH drafted the review. DW and AP corrected and reviewed the review. All authors contributed to the article and approved the submitted version.

## Conflict of Interest

The authors declare that the research was conducted in the absence of any commercial or financial relationships that could be construed as a potential conflict of interest.
